# COVID-19 Booster Vaccine Hesitancy among Hemodialysis Patients in Saudi Arabia Using the Health Belief Model: A Multi-Centre Experience

**DOI:** 10.3390/vaccines11010095

**Published:** 2022-12-31

**Authors:** Sami Alobaidi, Enad Alsolami, Abdalla Sherif, Mohammed Almahdy, Rady Elmonier, Waad Y. Alobaidi, Ahmed Akl

**Affiliations:** 1 Department of Internal Medicine, University of Jeddah, Jeddah 21493, Saudi Arabia; 2 Dr. Soliman Fakeeh Hospital, Jeddah 23323, Saudi Arabia; 3 Saudi German Hospital, Jeddah 21461, Saudi Arabia; 4 Internal Medicine Department, Alazhar University, Cairo 11651, Egypt; 5 International Medical Centre, Jeddah 21451, Saudi Arabia; 6 College of Medicine, Batterjee Medical College, Jeddah 21442, Saudi Arabia; 7 Urology and Nephrology Center, Mansoura University, Mansoura 35516, Egypt

**Keywords:** COVID-19 vaccination, intention to vaccinate, booster, CKD, hemodialysis, Saudi Arabia, health belief model

## Abstract

Objective: Vaccination hesitance for the COVID-19 booster dosage among hemodialysis patients is an important barrier in reducing morbidity and mortality linked to COVID-19 infection. Hence, this study aimed to explore the predictors of the third (booster) dose of COVID-19 vaccine intention among CKD patients on hemodialysis from the Kingdom of Saudi Arabia (KSA). Methods: This study was a multi-center cross-sectional study conducted at four dialysis centers in KSA from 13 February 2022 to 21 June 2022. The data was collected by the nephrologist in charge of the unit using a structured study questionnaire, which consisted of four parts; socio-demographic and clinical variables, questions about COVID-19 infection and subjective assessment of health state, COVID-19 booster dose vaccination intention and confidence in vaccines and preferences, and a health belief model. The study population consisted of 179 hemodialysis patients. Results: Participants in the study had conflicting health beliefs about their vulnerability to COVID-19 infection and the severity of the COVID-19 infection. Study participants expressed positive health beliefs about the advantages of the COVID-19 booster dose, and reported less perceived obstacles in receiving the vaccine. The influence of cues on action among the study population was high. A total of 140 (78.2%) hemodialysis patients expressed their intention to receive the COVID-19 booster dose. Patients who reported poor health in the self-rating of their health status had a substantially higher definite intention to take the COVID-19 booster dose, according to the chi-square test (11.16, df = 3, *p* = 0.01). There was a significant association between the constructs in the HBM model and COVID-19 vaccine (booster) intention. Marital status (OR = 1.67, CI 1.07–2.58) was found to be the strongest predictors of a definite intention to receive a COVID-19 booster dose. Confidence in the locally manufactured vaccine (OR = 0.33, CI 0.17–0.60), education (OR = 0.62, CI 0.41–0.93), and rating of health status (OR = 0.43 CI 0.25–0.74) were the strongest significant correlates of having no definite intention to take the COVID-19 vaccination. Conclusions: HBM constructs were found to be significantly associated with vaccination intention, which can be considered while planning policies to promote COVID-19 booster vaccination among hemodialysis patients. The study results could be utilized in drafting policies to improve COVID-19 booster dose vaccination uptake among hemodialysis population.

## 1. Introduction

The coronavirus disease 2019 (COVID-19) pandemic significantly affected individuals suffering from chronic medical conditions, such as chronic kidney disease (CKD), in terms of risk of infection, morbidity, and mortality [1]. Individuals suffering from CKD were reported to be at high risk of COVID-19 infection due to their dependence on in-center dialysis treatment, which is a high-risk environment for the transmission of COVID-19 infection [2]. Studies have also consistently shown that patients with CKD on hemodialysis carry an elevated risk of morbidity and mortality from COVID-19 [3,4,5]. According to a recent study, patients on hemodialysis have a high risk of dying after contracting COVID-19, with case fatalities at 28 days being recorded at 23.9% [6].

The vaccines against COVID-19 have proved their efficacy in reducing COVID-19 transmission and mitigating the severity of COVID-19 infection in the general population [7]. However, the efficacy and the serological response to vaccination against COVID-19 among the dialysis population have been shown to be variable [6]. Studies have revealed that patients with CKD are likely to respond to COVID-19 mRNA vaccinations less favorably than the general population [8]. According to a recent study, ChAdOx1 (Oxford-AstraZeneca) vaccines induce fewer neutralizing antibodies to the Delta VOC than BNT162b2 vaccines in patients receiving hemodialysis after vaccination (Pfizer–BioNTech) [9]. Among CKD patients, individuals with a previous transplant, those on concurrent immunosuppressive medication, and those on hemodialysis compared with peritoneal dialysis, were shown to be having significantly less seroconversion to COVID-19 vaccination [10]. According to a recent study, two doses of the COVID-19 vaccination were only 33% effective at preventing infection and 38% effective at preventing hospitalization in hemodialysis patients [6]. Another national registry study showed that in spite of having two doses of mRNA vaccine, 11% of fully vaccinated hemodialysis patients died following COVID-19 infection [11]. The aforementioned study’s findings make it clear that a primary immunization course of two doses does not adequately protect patients undergoing hemodialysis. Therefore, a third, or “booster”, dosage of the COVID-19 vaccine was recommended to further raise the immunogenicity of hemodialysis patients [12].

Despite the significant achievements in COVID-19 vaccine research, vaccine hesitancy still remains a significant global issue. Vaccine hesitancy is defined as a delay in accepting or refusing vaccines notwithstanding the availability of vaccination services [13]. Vaccination reluctance for the COVID-19 booster dosage uptake is a substantial barrier to reducing morbidity and mortality linked to COVID-19 infection, in particular among vulnerable communities. Previous published reports have indicated significant vaccine hesitancy among vulnerable patient groups with various chronic conditions [14,15,16]. Studies conducted among CKD patients also reported high levels of COVID-19 vaccine hesitancy [17]. A study conducted among hemodialysis patients in Egypt reported COVID-19 vaccine hesitancy among 41.7% of the study participants [18]. However, few studies have examined CKD patients’ resistance to receiving the COVID-19 booster dose. Only 70.29% of research participants with CKD were willing to get a booster dose of the COVID-19 vaccination, according to a recent Chinese study [19].

The health belief model (HBM) is one of the most widely utilized models to explore vaccination beliefs and vaccination intentions. Many previous studies have used HBM constructs to study COVID-19 vaccination intentions among various population groups. Though there are studies exploring COVID-19 vaccination among general population and healthcare workers using HBM in the Kingdom of Saudi Arabia (KSA), there have been no studies exploring COVID-19 vaccine hesitancy using HBM among any disease population from KSA [20].

To the best of our knowledge, no studies have yet been conducted to determine if CKD patients from Middle Eastern nations who are receiving hemodialysis would be willing to receive a COVID-19 booster dose. This study aimed to explore the sociodemographic and HBM predictors of the third (booster) dose of COVID-19 vaccine intention among CKD patients on hemodialysis from the Kingdom of Saudi Arabia (KSA).

## 2. Methodology

### 2.1. Study Design and Participants

The present study was a cross-sectional study using purposive sampling techniques to recruit participants. This study was conducted at four dialysis centers in KSA from 13 February 2022 to 21 June 2022, and a nephrologist in charge of the units conducted face-to-face interviews with the patients. The sample size was calculated based on a previous study conducted among maintenance hemodialysis patients in Egypt [18]. This study received permission from the institutional ethical committee with approval code 2022-03-193.

The questionnaire was adapted from a study investigating factors associated with healthcare workers’ intention to receive a COVID-19 booster dosage in Saudi Arabia (questionnaire in Supplementary File S1). The individuals were made aware that their participation was optional, and each one provided written informed consent. Patients receiving hemodialysis at the four dialysis centers were included in the study and those not willing to give consent for the study or those who were unable to understand the study question were excluded.

### 2.2. Instruments

The study questionnaire consisted of four sections. The initial part of the questionnaire contained questions related to socio-demographic and clinical variables, such as age, gender, marital status, education level, occupation, hemodialysis center, the cause of CKD, co-morbidities, and dialysis duration. The next portion included questions about COVID-19 infection for the respondent and/or close family members, as well as a subjective assessment of their health state on a five-point Likert scale ranging from very good to very poor. Regarding the intention to receive the COVID-19 booster dosage and preferences for locally or internationally produced COVID-19 vaccines, the questions in the following section gathered replies. On a four-point scale, immunization intent and trust in locally or internationally produced COVID-19 vaccines were evaluated. In light of the COVID-19 illness and booster vaccine, the final section of the questionnaire was based on the construct of the health belief model (HBM) [21]. In order to investigate the study participants’ beliefs regarding their susceptibility to COVID-19 infection, their susceptibility to the severe COVID-19 infection, the advantages of receiving a COVID-19 booster vaccine, the barriers to receiving a COVID-19 booster vaccine, and the cues that facilitate receiving a COVID-19 booster vaccine, HBM constructs were added to the study questionnaire. The following HBM constructs were included in this study: perceived susceptibility, perceived severity, perceived benefits, perceived barriers, and cues to actions. A four-point scale (strongly agree, agree, disagree, and strongly disagree) was used to rate participants agreement with statements.

## 3. Statistical Analysis

The Statistical Package for Social Sciences (SPSS Inc., Chicago, IL, USA, version 26.0 for Windows) was used to analyze the data. To examine the sociodemographic and clinical characteristics of the study participants, descriptive statistical analysis was used. Using chi-square tests and the independent sample *t*-test, the statistically significant relationships between the variables were examined. The sociodemographic and HBM predictors of COVID-19 booster dose uptake were analyzed using binary logistic regression; the intention to receive COVID-19 booster dose (1—intends to get vaccinated, 0—does not intend to get vaccinated) was defined as the dependent variable. In order to calculate odds ratios (OR) and 95% confidence intervals (CI), the forward LR method of regression was utilized. The level of statistical significance was maintained at *p* < 0.05.

## 4. Results

Demographics: The study received 179 complete responses; 60.9% of the study participants were males, 66.5% were Saudi nationals, 54.2% had studied up to high school level, and 65.9% were married. The mean age of the study population was 60.14 ± 13.86 years (ranging from 26 to 92 years). The study participants included retired workers (30.2%), house wives (31.3%), private sector employees (20.1%), self-employed (7.8%), government sector employees (5%), military sector (1.1%), unemployed (1.1%), students (1.1%), and workers in other sectors (2.2%). The major causes of CKD in the study population included diabetes (46.4%), hypertension (37.4%), unknown causes (6.1%), glomerulonephritis (3.9%), liver failure (4.5%), and hereditary (1.7%) causes. A total of 27.4% of the study population reported a history of COVID-19 infection in the past. A total of 48.6% reported a family history of COVID-19 infection. A subjective health state of 45.8% of the survey participants was judged as good. The median value of serum creatinine was 1.32 ± 0.36. The demographics of the study participants are shown in Table 1.

### 4.1. Health Beliefs among the Hemodialysis Patient Population

Participants in the study had conflicting health opinions about their vulnerability to COVID-19 infection. Only 40.2% of respondents in the study believed that they had a higher likelihood of contracting COVID-19 infection, and 51.9% said they were not concerned about it. Despite the lower susceptibility beliefs, 82.7% still believed that catching COVID-19 infection was a possibility for them.

The opinions of the study population on the severity of the COVID-19 infection were divided. Only 49.7% of survey participants felt that COVID-19-related problems were substantial, and only 38% agreed that getting infected with COVID-19 would make them seriously ill. A total of 56.4% of respondents indicated their worry of contracting a COVID-19 infection, despite their lower severity beliefs.

Participants in the study expressed good health perceptions about the advantages of the COVID-19 booster dose. A total of 76% of study participants agreed that receiving a COVID-19 booster dose could lessen their concern about contracting the disease, and 83.2% agreed that receiving a COVID-19 booster dose lessens their susceptibility to COVID-19 infection and its sequelae.

The participants reported less perceived obstacles to receiving the COVID-19 booster shot. Only 33% of the study participants expressed their concern with the possibility of side effects following COVID-19 booster dose vaccination; only 20.1% reported their concerns with the efficacy of the COVID-19 booster dose vaccination; only 15.6% reported concerns regarding the safety of the vaccine; and only 10.6% reported concerns with faulty/fake COVID-19 booster dose vaccination.

The study found a high influence of cues on action in the study participants. Among the study participants, 82.1% reported that adequate information would influence their decision-making in accepting the COVID-19 booster dose, and 83.2% stated that they would only take it if numerous people receiving hemodialysis also took the booster dose.

### 4.2. Confidence in Vaccines and Preferences

A total of 58.1% of those surveyed showed faith in the locally produced COVID-19 vaccine; 86% of respondents expressed faith in imported COVID-19 vaccinations. A total of 10.6% of respondents preferred locally made vaccines and 39.1% preferred imported vaccines. More chose locally produced vaccinations, which were statistically and significantly associated with vaccination preference and confidence in local vaccines (131.95, df 6, *p* 0.001). Vaccine preference and trust in foreign-made vaccines were shown to be significantly correlated (46.46, df 6, *p* 0.001), with a higher proportion of respondents expressing a preference for foreign-made vaccines.

Foreign-made vaccines were significantly preferred by non-Saudis (13.29, df = 2, *p* = 0.001) and people who self-reported having a low health status (12.75, df = 6, *p* = 0.047). The findings are summarized in Table 1.

### 4.3. COVID-19 Booster Vaccination Intent

Among the respondents, 140 (78.2%) expressed their intention to receive a COVID-19 booster dose: 25.7% reported definitely yes, and 52.5% reported probably yes. A total of 14% of participants responded that they were probably not intending to receive the COVID-19 booster dose, and 7.8% expressed definite intention not to receive a COVID-19 booster dose.

Patients who reported poor health in the self-rating of health status had a substantially higher definite intention to take the COVID-19 booster dose, according to the chi-square test (11.16, df = 3, *p* = 0.01). Table 2 provides a summary of the results. There was a significant association between the constructs in the HBM model and COVID-19 vaccine (booster) intention. The findings are summarized in Table 3.

In the binary logistic regression analysis, marital status (OR = 1.67, CI 1.07–2.58) was the strongest predictor of a definite intention to receive a COVID-19 booster dose. Confidence in the locally manufactured vaccine (OR = 0.33, CI 0.17–0.60), education (OR = 0.62, CI 0.41–0.93), and rating of health status (OR = 0.43 CI 0.25–0.74) were the strongest significant correlates of having no definite intention to take the COVID-19 vaccination. There was no statistically significant predictors of HBM constructs influencing the COVID-19 vaccination booster dose uptake, and the Hosmer-Lemeshow goodness-of-fit test showed poor fit in binary logistic regression with HBM variables. The findings are summarized in Table 4.

## 5. Discussion

In this study on COVID-19 booster vaccination willingness in patients with chronic kidney disease, it was discovered that, overall, 78.2% of respondents stated their intention to receive a COVID-19 vaccine booster, and 25.7% reported a definite intention. A recent study survey from China among 350 CKD patients showed that around 70.29% of the respondents reported their willingness to receive a COVID-19 booster vaccine dose [19]. When compared to a recent study conducted among patients with another chronic medical condition (inflammatory bowel disease) in Kuwait that reported only 58.1% acceptance, the willingness to receive a COVID-19 vaccination booster dose was higher in our study [22]. Another study among healthcare workers in SA found that 71.1% of study participants reported positive intent for receiving a COVID-19 booster dose, which is lower than in our study [20]. However, the rate of acceptance for COVID-19 booster vaccine doses in this study was similar or lower when compared to many studies conducted among the general population in various countries. A recent study in China among the adult general population found that 76.8% of participants were willing to take the COVID-19 booster vaccine dose [23]. Another study among the Pakistani public reported 77.8% booster dose willingness [24]. A comparable favorable intention was stated by 84.5% of respondents in a recent cross-sectional study among Japanese medical students [25]. Another study among university students and staff in Germany found an acceptance rate of 87.8% [26].

The study participants exhibited less anxiety about their vulnerability to COVID-19 infection and their risk of contracting COVID-19 infection among HBM factors. The full vaccination status and lower rate of infection in the country at the time of the study could be the reason for reporting low perceived susceptibility among study participants. Considering the variable efficacy of the two doses of COVID-19 vaccination among CKD patients on hemodialysis, the low perceived susceptibility could be dangerous as it might lead to less compliance with COVID-19 preventive measures and incidence of serious infection in this vulnerable population [27]. Public health awareness campaigns should be planned to educate CKD patients regarding their susceptibility to COVID-19 infection in spite of two doses of COVID-19 vaccination. A majority of the participants agreed with the possibility of getting COVID-19 infection and expressed fear of COVID-19 infection. However, they perceived the severity and complications of COVID-19 infection as less severe. The lower level of perceived severity of COVID-19 infection in the study participants indicates less knowledge and awareness concerning studies reporting higher morbidity and mortality in COVID-19-infected patients with CKD on hemodialysis, even after taking two doses of COVID-19 vaccination [28,29]. There is a need to educate CKD patients on hemodialysis regarding their higher susceptibility to serious complications following COVID-19 infection so that they seriously follow preventive measures against COVID-19 infection. A significant proportion of respondents perceived the COVID-19 booster dose as beneficial in reducing their susceptibility to COVID-19 infection, which is an encouraging finding. The majority of participants reported less barriers to getting the COVID-19 booster shot and agreed that it was safe, efficient, and side-effect-free immunization. Such positive perception regarding the safety and effectiveness of the COVID-19 vaccine can increase vaccination intentions [20]. Many of the study’s participants noted the importance of having accurate information about the COVID-19 booster dosage vaccine and the vaccination uptake by numerous other dialysis patients, as well as the influence of signals in decision-making on the uptake of the COVID-19 booster shot. Considering the low barrier to COVID-19 booster dose uptake and the importance of cues, there is an urgent need to conduct educational campaigns targeting dialysis patients to provide adequate information regarding the rationale, effectiveness, and safety of the COVID-19 booster dose. Moreover, vaccination centers can be setup in dialysis facilities so that patients can witness COVID-19 booster uptake by other hemodialysis patients.

This study also found marital status to be an important predictor of definite intention for COVID-19 booster uptake. A recent study among healthcare workers from KSA also reported significant association between a definite intention to receive a booster dose and marital status [20]. A recent study also reported more awareness among married individuals regarding the COVID-19 vaccine. The higher rate of COVID-19 booster dose acceptance among married individuals might be due to higher awareness and the perception of risk to their spouse [30]. However, confidence in the locally manufactured vaccine, education, and rating of health status were found to be significantly associated with having no definite intention to take the COVID-19 vaccination. Italian and French research of dialysis patients has found a substantial correlation between vaccine hesitation and a younger age, a lower impression of disease severity and vaccine efficacy, and a lower level of trust in the vaccination, the medical system, and scientists [17]. The absence of a locally made vaccine and a lower perception of disease severity due to higher health status might have contributed to vaccine hesitancy.

The study results have significant policy implications. Significant sections of the hemodialysis population had at least a positive intention to receive a COVID-19 booster dose. Policies should be made to translate positive intentions into actual vaccination uptake behavior by proactively reaching out to hemodialysis patients, preferably by conducting vaccination at the dialysis facility itself whenever possible. This study also found that healthy dialysis patients had significant vaccine hesitancy. There is a need to conduct educational programs among the dialysis population to increase awareness about the risk of COVID-19 infection and its complications among patients with CKD.

While interpreting the findings, it is important to consider the limitations of the current study. The sample size was small. The intention to acquire a COVID-19 booster dose in this study might not accurately reflect actual vaccination uptake behavior as a number of other factors, such as accessibility and co-morbid medical and psychological problems, affect vaccination uptake. Finally, this study was conducted in four selected dialysis centers and the findings from this study cannot be generalized to all patients on hemodialysis in KSA.

## 6. Conclusions

Overall, 78.2% of the respondents to this survey stated that they intended to receive the COVID-19 booster dose. HBM constructs were found to be significantly associated with vaccination intention, which can be considered while planning policies to promote COVID-19 booster vaccination among hemodialysis patients.

## Figures and Tables

**Table 1:** Association between vaccine preferences and sociodemographic variables.

Variable						
		Vaccine Preference			*p*-Value	
		Participants	Local	Foreign-Made	No Preference	
Gender						
	Male	109 (60.9%)	11 (10.1%)	41 (37.6%)	57 (52.3%)	0.796
	Female	70 (39.1%)	8 (11.4%)	29 (41.4%)	33 (47.1%)	
Nationality						
	Saudi	119 (66.5%)	19 (16%)	39 (32.8%)	61 (51.3%)	0.001
	Non-Saudi	60 (33.5%)	0	31 (51.7%)	29 (48.3%)	
Education						
	Nil	27 (15.1%)	3 (11.1%)	7 (25.9%)	17 (63%)	
	High school	97 (54.2%)	11 (11.3%)	37 (38.1%)	49 (50.5%)	0.629
	Bachelor	50 (27.9%)	5 (10%)	23 (46%)	22 (44%)	
	Masters/PhD	5 (2.8%)	0	3 (60%)	2 (40%)	
Occupation						
	Self employed	14 (7.8%)	3 (21.4%)	3 (21.4%)	8 (57.1%)	0.330
	Private sector	36 (20.1%)	1 (2.8%)	18 (50%)	17 (47.2%)	
	Government sector	9 (5%)	1 (11.1%)	1 (11.1%)	7 (77.8%)	
	Military sector	2 (1.1%)	1 (50%)	0	1 (50%)	
	House wife	56 (31.3%)	6 (10.7%)	23 (41.1%)	27 (48.2%)	
	Retired	54 (30.2%)	6 (11.1%)	20 (37%)	28 (51.9%)	
	Unemployed	2 (1.1%)	0	1 (50%)	1 (50%)	
	Student	2 (1.1%)	0	2 (100%)	0	
	Others	4 (2.2%)	1 (25%)	2 (50%)	1 (25%)	
Marital Status						0.145
	Married	118 (65.9%)	10 (8.5%)	49 (41.5%)	59 (50%)	
	Single	11 (6.1%)	1 (9.1%)	7 (63.6%)	3 (27.3%)	
	Widowed	50 (27.9%)	8 (16%)	14 (28%)	28 (56%)	
Past history of COVID-19 infection						
	Yes	49 (27.4%)	6 (12.2%)	20 (40.8%)	23 (46.9%)	0.831
	No	130 (72.6%)	13 (10%)	50 (38.5%)	67 (51.5%)	
Family history of COVID-19 infection						
	Yes	87 (48.6%)	9 (10.3%)	37 (42.5%)	41 (47.1%)	0.653
	No	92 (51.4%)	10 (10.9%)	33 (35.9%)	49 (53.3%)	
Self rating of Health status						
	Very good	44 (24.6%)	10 (22.73%)	13 (29.55%)	21 (47.73%)	0.047
	Good	82 (45.8%)	8 (9.76%)	35 (42.68%)	39 (47.56%)	
	Fair	46 (25.7%)	1 (2.17%)	18 (39.13%)	27 (58.70%)	
	Poor	7 (3.9%)	0	4 (57.14%)	3 (42.86%)	

**Table 2:** Association between sociodemographic and clinical variables and COVID-19 vaccine third dose (booster) intention.

Variable				
		COVID-19 Vaccine (Booster) Intention		
		DefinitelyYes	Probably Yes/ProbablyNo/Definitely No	*p*-Value
Gender				
	Male	24 (22%)	85 (78%)	0.160
	Female	22 (31.4%)	48 (68.6%)	
Nationality				
	Saudi	33 (27.7%)	86 (72.3%)	0.381
	Non-Saudi	13 (21.7%)	47 (78.3%)	
Marital status				
	Married	27 (22.9%)	91 (77.1%)	0.270
	Single	2 (18.2%)	9 (81.8%)	
	Widowed/divorced/separated	17 (34%)	33 (66%)	
Education				
	Nil	5 (18.5%)	22 (81.5%)	0.330
	High school and below	29 (29.9%)	68 (70.1%)	
	Bachelor or diploma	12 (24%)	38 (76%)	
	Master and PhD	0	5 (100%)	
Self rating of health status				
	Very good	18 (40.9%)	26 (59.1%)	0.011
	Good	22 (26.8%)	60 (73.2%)	
	Fair	5 (10.9%)	41 (89.1%)	
	Poor	1 (14.3%)	6 (85.7%)	

**Table 3:** Association between HBM variables and COVID-19 vaccine third dose (booster) intention.

Variable					
		Total Responses	COVID-19 Vaccine (Booster) Intention		
			DefinitelyYes	Probably Yes/ProbablyNo/Definitely No	*p*-Value
Perceived susceptibility of contracting COVID-19					
My chance of getting COVID-19 in the next few months is great					
	Strongly Agree				0.012
	Agree	72 (40.2%)	18 (25%)	54 (75%)	
	Disagree	104 (58.1%)	25 (24%)	79 (76%)	
	Strongly Disagree	3 (1.7%)	3 (6.5%)	0	
I am worried about the likelihood of getting COVID-19					
	Strongly Agree	2 (1.1%)	1 (50%)	1 (50%)	0.016
	Agree	84 (46.9%)	15 (17.9%)	69 (82.1%)	
	Disagree	91 (50.8%)	28 (30.8%)	63 (69.2%)	
	Strongly Disagree	2 (1.1%)	2 (100%)	0	
Getting COVID-19 is currently a possibility for me					
	Strongly Agree	2 (1.1%)	0	2 (100%)	0.007
	Agree	146 (81.6%)	31 (21.2%)	115 (78.8%)	
	Disagree	30 (16.8%)	14 (46.7%)	16 (53.3%)	
	Strongly Disagree	1 (0.6%)	1 (2.2%)	0	
Perceived Severity					
Complications from COVID-19 are serious					
	Strongly Agree	15 (8.4%)	10 (66.7%)	5 (33.3%)	<0.001
	Agree	74 (41.3%)	27 (36.5%)	47 (63.5%)	
	Disagree	89 (49.7%)	8 (9%)	81 (91%)	
	Strongly Disagree	1 (0.6%)	1 (2.2%)	0	
I will be very sick if I get COVID-19					
	Strongly Agree	10 (5.6%)	5 (50%)	5 (50%)	0.023
	Agree	58 (32.4%)	9 (15.5%)	49 (84.5%)	
	Disagree	110 (61.5%)	31 (28.2%)	79 (71.8%)	
	Strongly Disagree	1 (0.6%)	1 (2.2%)	0	
I am afraid of getting COVID-19					
	Strongly Agree	5 (2.8%)	3 (60%)	2 (40%)	0.007
	Agree	96 (53.6%)	18 (18.8%)	78 (81.3%)	
	Disagree	76 (42.5%)	23 (30.3%)	53 (69.7%)	
	Strongly Disagree	2 (1.1%)	2 (100%)	0	
Perceived benefits of COVID-19 vaccination					
Third (booster) dose of COVID-19 Vaccine is a good idea because I feel less worried about catching COVID-19					
	Strongly Agree	7 (3.9%)	5 (71.4%)	2 (28.6%)	0.006
	Agree	129 (72.1%)	36 (27.9%)	93 (72.1%)	
	Disagree	42 (23.5%)	5 (11.9%)	37 (88.1%)	
	Strongly Disagree	1 (0.6%)	0	1 (100%)	
Receiving third (booster) dose of COVID-19 Vaccine decreases my chance of getting COVID-19 or its complications					
	Strongly Agree	7 (3.9%)	5 (71.4%)	2 (28.6%)	0.001
	Agree	142 (79.3%)	39 (27.5%)	103 (72.5%)	
	Disagree	30 (16.8%)	2 (6.7%)	28 (93.3%)	
	Strongly Disagree				
Perceived barriers of COVID-19 vaccination					
Worry that possible side-effects of the third (booster) dose COVID-19 vaccine would interfere with my usual activities					
	Strongly Agree	8 (4.5%)	0	8 (100%)	<0.001
	Agree	51 (28.5%)	4 (7.8%)	47 (92.2%)	
	Disagree	117 (65.4%)	40 (34.2%)	77 (65.8%)	
	Strongly Disagree	3 (1.7%)	2 (66.7%)	1 (33.3%)	
I am concerned about the efficacy of the third (booster) dose COVID-19 vaccine					
	Strongly Agree	2 (1.1%)	1 (50%)	1 (50%)	0.003
	Agree	34 (19%)	2 (5.9%)	32 (94.1%)	
	Disagree	141 (78.8%)	41 (29.1%)	100 (70.9%)	
	Strongly Disagree	2 (1.1%)	2 (100%)	0	
I am concerned about the safety of the third (booster) dose COVID-19 vaccine					
	Strongly Agree	2 (1.1%)	0	2 (100%)	0.007
	Agree	26 (14.5%)	3 (11.5%)	23 (88.5%)	
	Disagree	148 (82.7%)	40 (27%)	108 (73%)	
	Strongly Disagree	3 (1.7%)	3 (100%)	0	
I am concerned of the faulty/fake COVID-19 vaccine					
	Strongly Agree	1 (0.6%)	0	1 (100%)	<0.001
	Agree	18 (10.1%)	6 (33.3%)	12 (66.7%)	
	Disagree	154 (86%)	34 (22.1%)	120 (77.9%)	
	Strongly Disagree	6 (3.4%)	6 (100%)	0	
Cues to action					
I will only take the third (booster) dose COVID-19 vaccine if I was given adequate information about it					
	Strongly Agree	6 (3.4%)	4 (66.7%)	2 (33.3%)	0.002
	Agree	141 (78.8%)	41 (29.1%)	100 (70.9%)	
	Disagree	24 (13.4%)	1 (4.2%)	23 (95.8%)	
	Strongly Disagree	8 (4.5%)	0	8 (100%)	
I will only take the third (booster) dose COVID-19 vaccine if the vaccine is taken by many healthcare workers in Saudi Arabia					
	Strongly Agree	7 (3.9%)	5 (71.4%)	2 (28.6%)	0.003
	Agree	142 (79.3%)	39 (27.5%)	103 (72.5%)	
	Disagree	22 (12.3%)	2 (9.1%)	20 (90.9%)	
	Strongly Disagree	8 (4.5%)	0	8 (100%)	

**Table 4:** Predictors of definite COVID-19 vaccine third dose (booster) uptake (binary logistic regression analysis).

Variable	OR (Exp B)	Wald	df	*p*	95% Con Dence Interval	
Constant	22.77	10.34	1	0.001	Lower limit	Upper limit
Confidence in the locally manufactured vaccine	0.328	12.74	1	<0.001	0.178	0.605
Education	0.623	5.31	1	0.021	0.416	0.932
Marital Status	1.665	5.12	1	0.023	1.071	2.588
Rating of Health Status	0.438	9.46	1	0.002	0.258	0.741

## Data Availability

The data presented in this study are available on request from the corresponding author. The data are not publicly available due to privacy concerns.

## Supplementary Materials

The following supporting information can be downloaded at: https://www.mdpi.com/article/10.3390/vaccines11010095/s1, questionnaire.
