# Supplementary material for: COVID-19 Booster Vaccine Hesitancy among Hemodialysis Patients in Saudi Arabia Using the Health Belief Model: A Multi-Centre Experience
Source: Vaccines (Basel). 2022 Dec 31;11(1):95. doi: 10.3390/vaccines11010095 (PMC9862905; doi:10.3390/vaccines11010095)
Supplement: Supplementary file 1 [file vaccines-11-00095-s001.zip › vaccines-2059960-supplementary.pdf]

Predictors of Intent to Receive the booster dose of COVID-19 Vaccine among hemodialysis patients in Saudi Arabia: A Survey Study/مدى تقبل مرضى غسيل الكلى الدموي في السعودية لتلقي جرعة التطعيم المعززة ضد فيروس كورونا

You need to ask the question based on the patient's vaccine status:
1-if received 3 doses: ask about future booster dose if recommended by MOH
2-if received 2 doses: ask about 3rd dose
3-if received1 dose only: ask about second and third dose
4-If received no doses: ask about first dose

* Required

1. I agree on participation and I am aged 16 years old or more/أوافق على المشاركة وعمري 16 سنة أو أكثر *

Mark only one oval.

- Yes/نعم
- No/لا

2. Have you been vaccinated for COVID-19/ هل تم تطعيمك ضد فيروس كورونا المستجد *

Mark only one oval.

- a. Yes, completed 3 doses/نعم وتلقيت 3 جرعات
- b. Yes, completed 2 doses/نعم وتلقيت جرعتين
- c. Yes, received one dose only/نعم وتلقيت جرعة واحدة فقط
- d. No/لا

3. From where do you receive information about the COVID19 vaccines (Select all that apply)?(من اي مصدر تلقيت معلومات عن اللقاح ضد فيروس كورونا المستجد(اختار كل ماينطبق *

Check all that apply.

- [ ] 1. Dialysis staff (nurses or technicians)
- [ ] 2. My kidney doctor
- [ ] 3. Other patients on dialysis
- [ ] 4. Friends and family
- [ ] 5. TV news
- [ ] 6. Newspapers
- [ ] 7. Social media (Facebook, twitter, Instagram, etc)
- [ ] 7. I have not received any information about COVID19 vaccines
- [ ] Other: ____________________

Section A: GENERAL INFORMATION/ معلومات عامة

4. 1. Age in years / العمر بالسنوات *

____

5. 2. Gender /الجنس *

Mark only one oval.

- ( ) a. Male/ذكر
- ( ) b. Female/ أنثى

6. 3. Nationality / الجنسية *

Mark only one oval.

- ( ) a. Saudi/سعودي
- ( ) b. Non Saudi/غير سعودي

7. 4. Marital Status / /الحالة الإجتماعية *

Mark only one oval.

- a. Married/متزوج
- Widow/divorced/seperated/منفصل/مطلق/أرمل
- Single/never married/ لم يتزوج قط / أعزب

8. 5. Highest education /التعليم *

Mark only one oval.

- a. High school and below/ تعليم ثانوي أو أقل
- b. Bachelor or diploma/ بكالوريوس او دبلوم
- c. Master or PhD/ ماجستير أو دكتوراه
- d. No Education/لم ادرس

9. 6. Occupation / المهنة *

Mark only one oval.

- Self-employed/أعمال حرة
- Employee in private sector/موظف في القطاع الخاص
- Employee in government office/موظف في القطاع حكومي
- Military sector/القطاع العسكري
- Housewife/ربه منزل
- Retired/متقاعد
- Unemployed/عاطل عن العمل
- Student/طالب
- Others/اخرى

10. 7. Hemodialysis Center/مركز الغسيل *

Mark only one oval.

- DSFH
- IMC
- SGH
- Other: ____________

11. MRN *

____

12. 8. The cause of ESRD? سبب الفشل الكلوي *

Mark only one oval.

- a. Diabetes Mellites/السكري
- b. Hypertension/ارتفاع ضغط الدم
- c. Hereditary kidney disease/مرض وراثي
- d. Glomerulonephritis/التهاب كبيبات الكلى
- e. Unknown/غير معروف
- Other: ____________

13. 9. Co-morbidities (Select all that apply) / (الأمراض)اختار كل ماينطبق المزمنة *

Check all that apply.

- [ ] a. السكري / Diabetes Mellitus
- [ ] b. ارتفاع ضغط الدم/ Hypertension
- [ ] c. امراض القلب / Cardiac disease
- [ ] d. CVA/جلطات المخ
- [ ] e. chronic lung disease/امراض صدرية مزمنة
- [ ] Other: ____________

14. 10. Vascular access/طريقة وصلة الغسيل *

Mark only one oval per row.

	Arterio-venous fistula	Arterio-venous graft	Central venous catheter
**الوصلة المبدئية/At the start of dialysis**	⬭	⬭	⬭
**الوصلة الحالية/ Current access**	⬭	⬭	⬭

15. 11. Latest Kt/V in the last 3 months/ في الثلاث الأشهر (Kt/V) آخر معدل لكفاءة الغسيل الماضية *

Mark only one oval.

- Not available/ غير متوفر
- Other: ____________________

16. 13. Years on dialysis/عدد سنوات الغسيل *

Mark only one oval.

- a. less than a year/اقل من سنة
- b. 1-3 years/من سنة الى 3 سنوات
- c. More than 3 years/اكثر من 3 سنوات

Previous COVID-19 illness/(فيروس كورونا المستجد) 19- الإصابة بكوفيد

17. 12. Have you ever been ill with COVID-19 in the past? هل سبق ان أصبت بفيروس كورونا المستجد؟ *

Mark only one oval.

- a. Yes/ نعم
- b. No/لا

18. 13. Has any of your close family members been ill with COVID-19? هل أصيب أحد أفراد أسرتك المقربين بفيروس كورونا المستجد؟ *

Mark only one oval.

- a. Yes/ نعم
- b. No/لا

General Health/الصحة العامة

19. 15. How do you rate your overall health?كيف تقيم صحتك العامة؟

Mark only one oval.

- Very good/جيدة جدا
- Good/جيدة
- Fair/ مقبولة
- Poor/سيئة
- Very poor/سيئة جدا

Section B: ACCEPTABILITY OF COVID-19 VACCINATION . تقبل التطعيم ضد فيروس كورونا المستجد

You need to ask the question based on patient's vaccine status:
1-if received 3 doses: ask about future booster dose if recommended by MOH
2-if received 2 doses: ask about 3rd dose
3-if received1 dose only: ask about second and third dose
4-If received no doses: ask about first dose

20. 16. The medical experts recommend to take the booster dose of COVID-19 vaccine for hemodialysis patients, Are you planning to take it ?أوصى الخبراء الطبيين بأخذ جرعة التطعيم المعززة لمرضى الغسيل الدموي ضد فيروس كورونا, هل تخطط لأخذها ؟ *

Mark only one oval.

- a. Definitely no/قطعا لا
- b. Probably no/على الأرجح لا
- c. Possibly yes/ربما نعم
- d. Definitely yes/قطعا نعم

21. 17. Rate your confidence in using locally manufactured COVID-19 vaccine: في حال تم تصنيع لقاح محلي لفيروس كورونا المستجد، قيم ثقتك في استخدام هذا اللقاح؟ *

Mark only one oval.

- a. Completely confident/ واثق تماما
- b. Confident/ واثق
- c. Not confident/ غير واثق
- d. Completely not confident/غير واثق تماما

22. 18. Rate your confidence in using foreign-manufactured (imported) COVID-19 vaccine:. قيم ثقتك في استخدام لقاح أجنبي الصنع (مستورد) لفيروس كورونا المستجد *

Mark only one oval.

- a. Completely confident/ واثق تماما
- b. Confident/ واثق
- c. Not confident/ غير واثق
- d. Completely not confident/غير واثق تماما

23. 19. Please indicate your preferences in local/imported COVID-19 vaccine: يرجى تحديد اللقاح المفضل لديك *

Mark only one oval.

- a. I prefer local manufactured COVID-19 vaccine/أنا أُفَضِّلْ لقاح محلي الصنع
- b. I prefer imported/foreign manufactured COVID-19 vaccine أنا أُفَضِّلْ لقاح مستورد (أجنبي الصنع))
- c. I don't have preference as long as there is vaccine available/ليس لدي أفضلية طالما كان هناك لقاح متوفر

Section D: HEALTH BELIEF MODEL نموذج الاعتقاد الصحي

Perceived susceptibility of contacting COVID-19 احتمالية الإصابة بفيروس كورونا المستجد المتوقعة

24. 20. My chance of getting COVID-19 in the next few months is great/امكانية إصابتي بفيروس كورونا المستجد في الأشهر القليلة المقبلة عالية *

Mark only one oval.

- a. Strongly agree/ موافق بشدة
- b. Agree/موافق
- c. Disagree/معارض
- d. Strongly disagree/معارض بشدة

25. 21. I am worried about the likelihood of getting COVID-19.أنا قلق من احتمال الإصابة بفيروس كورونا المستجد *

Mark only one oval.

- a. Strongly agree/ موافق بشدة
- b. Agree/موافق
- c. Disagree/معارض
- d. Strongly disagree/معارض بشدة

26. 22. Getting COVID-19 is currently a possibility for me. الإصابة بفيروس كورونا المستجد احتمال وارد بالنسبة لي *

Mark only one oval.

- a. Strongly agree/ موافق بشدة
- b. Agree/موافق
- c. Disagree/معارض
- d. Strongly disagree/معارض بشدة

Perceived severity of COVID-19. توقعات خطورة الإصابة بفيروس كورونا المستجد

27. 23. Complications of COVID-19 are serious. تعد مضاعفات فيروس كورونا المستجد خطيرة *

Mark only one oval.

- a. Strongly agree/ موافق بشدة
- b. Agree/موافق
- c. Disagree/معارض
- d. Strongly disagree/معارض بشدة

28. 24. I will be very sick if I get COVID-19. إذا أصبت لا سمح الله بفيروس كورونا المستجد، سأكون مريضًا جدًا (أعراض قوية) *

Mark only one oval.

- a. Strongly agree/ موافق بشدة
- b. Agree/موافق
- c. Disagree/معارض
- d. Strongly disagree/معارض بشدة

29. 25. I am afraid of getting COVID-19. أخشى من الإصابة بفيروس كورونا المستجد *

Mark only one oval.

- a. Strongly agree/ موافق بشدة
- b. Agree/موافق
- c. Disagree/معارض
- d. Strongly disagree/معارض بشدة

Perceived benefits of booster dose of COVID-19 vaccine. الفوائد المتوقعة من جرعة التطعيم المعززة ضد فيروس كورونا

30. 26.Booster dose of COVID-19 vaccine is a good idea because I will not have to worry about catching COVID-19. جرعة التطعيم المعززة فكرة جيدة لأنني لن أقلق بشأن الإصابة بفيروس كورونا المستجد *

Mark only one oval.

- a. Strongly agree/ موافق بشدة
- b. Agree/موافق
- c. Disagree/معارض
- d. Strongly disagree/معارض بشدة

31. 27. Receiving the booster dose of COVID-19 vaccine decreases my chance of getting COVID-19 or its complications. اخذ جرعة التطعيم المعززة ضد فيروس كورونا يقلل من احتمالية إصابتي بفيروس كورونا المستجد ومضاعفاته *

Mark only one oval.

- a. Strongly agree/ موافق بشدة
- b. Agree/موافق
- c. Disagree/معارض
- d. Strongly disagree/معارض بشدة

Perceived barriers of booster dose of COVID-19 vaccine. العوائق المتوقعة من تلقي جرعة التطعيم المعززة ضد فيروس كورونا

32. 28. The possible side-effects of the booster dose of vaccine would interfere with my usual activities. الآثار الجانبية المحتملة بعد تلقي جرعة التطعيم المعززة ستؤثر على حياتي اليومية *

Mark only one oval.

- a. Strongly agree/ موافق بشدة
- b. Agree/موافق
- c. Disagree/معارض
- d. Strongly disagree/معارض بشدة

33. 29. I am concerned about the efficacy of the booster dose of the vaccine. انا قلق من عدم فعالية جرعة التطعيم المعززة ضد فيروس كورونا *

Mark only one oval.

- a. Strongly agree/ موافق بشدة
- b. Agree/موافق
- c. Disagree/معارض
- d. Strongly disagree/معارض بشدة

34. 30. I am concerned about the safety of the booster dose of the vaccine. انا قلق بشأن سلامة جرعة التطعيم المعززة ضد فيروس كورونا (التطعيم غير آمن) *

Mark only one oval.

- a. Strongly agree/ موافق بشدة
- b. Agree/موافق
- c. Disagree/معارض
- d. Strongly disagree/معارض بشدة

35. 31. I am concerned about the faulty/fake COVID-19 vaccine. انا قلق بشأن تلقي لقاح مزيف او خاطئ (فاسد او غير فعال) ضد فيروس كورونا *

Mark only one oval.

- a. Strongly agree/ موافق بشدة
- b. Agree/موافق
- c. Disagree/معارض
- d. Strongly disagree/معارض بشدة

Cues to action. الدوافع لتلقي اللقاح

36. 32. I will only take the booster dose of COVID-19 vaccine if I was given adequate information about it. سوف أتلقى جرعة التطعيم المعززة إذا أعطيت معلومات كافية عنها *

Mark only one oval.

- a. Strongly agree/ موافق بشدة
- b. Agree/موافق
- c. Disagree/معارض
- d. Strongly disagree/معارض بشدة

37. 33. I will only take the booster dose of vaccine if the vaccine is taken by many dialysis patients. سوف أتلقى جرعة التطعيم المعززة إذا تم أخذ اللقاح من قبل الكثير من مرضى الغسيل *

Mark only one oval.

- a. Strongly agree/ موافق بشدة
- b. Agree/موافق
- c. Disagree/معارض
- d. Strongly disagree/معارض بشدة

This content is neither created nor endorsed by Google.

Google Forms
